# Infection risks associated with daratumumab-containing regimens in multiple myeloma: a systematic review and meta-analysis

**DOI:** 10.3389/fonc.2025.1729177

**Published:** 2026-01-06

**Authors:** Zeng-Yi Huang, Xiao-Lian Liu, Ting Li, Chao-Han Luo

**Affiliations:** 1 Faculty of Chinese Medicine, Macau University of Science and Technology, Macau, Macau SAR, China; 2 Department of Hematology, Gaozhou People’s Hospital, Maoming, Guangdong, China

**Keywords:** multiple myeloma, daratumumab, infection, pneumonia, meta-analysis, randomized controlled trials

## Abstract

Daratumumab, a CD38-targeting monoclonal antibody, is a key component of therapy for both newly diagnosed and relapsed or refractory multiple myeloma. By depleting CD38-expressing immune effector cells and reducing immunoglobulin levels, daratumumab may increase susceptibility to infections. To quantify this risk, we performed a systematic review and meta-analysis of randomized phase II and III trials comparing daratumumab-containing regimens with standard therapies in adults with multiple myeloma. Databases including PubMed, Embase, Web of Science, the Cochrane Library, and ClinicalTrials.gov were searched through 14 October 2025, following PRISMA 2020 guidelines. Nine trials encompassing 5,281 patients were included. Daratumumab-based regimens were associated with an increased risk of any infection (risk ratio [RR] 1.23; 95% confidence interval [CI] 1.14–1.33), grade ≥3 infection (RR 1.29; 95% CI 1.17–1.42), and pneumonia (RR 1.60; 95% CI 1.24–2.07). Subgroup analyses showed consistent results across disease stages and transplant eligibility groups. Infection-related mortality was uncommon (≤2%) and did not differ significantly between arms. These findings indicate that daratumumab-based therapy increases infection risk, particularly for severe infections and pneumonia, but the absolute mortality remains low. Proactive infection prevention and close clinical monitoring are warranted as the use of daratumumab continues to expand. This study was prospectively registered in PROSPERO (CRD420251165266).

**Systematic Review Registration:**

https://www.crd.york.ac.uk/PROSPERO/, CRD420251165266.

## 1 Introduction

Multiple myeloma (MM) is a malignant plasma cell disorder characterized by clonal proliferation within the bone marrow and secondary immunodeficiency due to impaired antibody production (1). Over the past decade, driven by the development of novel agents, the therapeutic landscape of MM has evolved substantially with the introduction of proteasome inhibitors, immunomodulatory drugs, and monoclonal antibodies (2–4). Among these, the anti-CD38 monoclonal antibody daratumumab has become a cornerstone of therapy for both newly diagnosed (NDMM) and relapsed/refractory MM (RRMM) (5–7).

Daratumumab exerts its antimyeloma activity through several mechanisms, including complement-dependent cytotoxicity, antibody-dependent cellular cytotoxicity, phagocytosis, and direct induction of apoptosis (8). However, CD38 is also expressed on immune effector cells such as natural killer (NK) cells, regulatory T cells, and subsets of B and T lymphocytes (9). Consequently, daratumumab may suppress immune surveillance and humoral immunity, leading to hypogammaglobulinemia and an increased susceptibility to infections (10–12). Clinical observations suggest that infections, particularly upper respiratory tract infections and pneumonia, are among the most common non-hematologic toxicities associated with daratumumab-based therapy (13, 14).

Although infection is a recognized adverse effect, the magnitude and consistency of this risk remain uncertain. Results from individual randomized controlled trials (RCTs) have been variable, likely due to differences in patient populations (NDMM vs. RRMM), treatment backbones, and definitions of infection endpoints (15, 16). Previous systematic reviews have primarily focused on overall safety outcomes or combined observational and interventional data, limiting the strength of inference (17, 18). To date, no comprehensive meta-analysis has specifically quantified infection risks based solely on randomized phase II/III evidence.

Given the expanding use of daratumumab across frontline and relapsed settings, an accurate estimation of infection risk is crucial to guide prophylactic and supportive care strategies. Therefore, we performed a systematic review and meta-analysis of RCTs to quantify the incidence and relative risk of any-grade infections, grade ≥3 infections, and pneumonia in patients receiving daratumumab-containing regimens compared with non-daratumumab–containing therapy. In addition, contemporary International Myeloma Working Group (IMWG) guidance emphasizes infection-prevention measures, including vaccination (influenza, pneumococcal, COVID-19), antiviral prophylaxis for herpes zoster during proteasome inhibitor or anti-CD38 exposure, selective antibacterial prophylaxis in high-risk periods, and intravenous immunoglobulin for recurrent or severe infections with hypogammaglobulinemia, highlighting the clinical relevance of quantifying daratumumab-associated infection risk (19).

## 2 Methods

### 2.1 Literature search

We systematically searched PubMed, Embase, Web of Science, the Cochrane Library, and the trial registry ClinicalTrials.gov from database inception to 14 October 2025 (20, 21). Search strategies combined controlled vocabulary (MeSH/Emtree) and free-text terms related to “daratumumab,” “multiple myeloma,” and “infection,” using Boolean and proximity operators; full strategies are provided in **Supplementary Table S1**. Eligibility was restricted to English-language full-text publications. We also screened reference lists of relevant trials and reviews (22). Records were deduplicated in reference-management software, and titles/abstracts and full texts were screened independently by two reviewers.

### 2.2 Eligibility criteria

Studies were selected according to the PICOS framework (23). Population: adults (≥18 years) with NDMM or RRMM. Intervention: daratumumab-containing regimens added to a standard backbone. Comparison: the same backbone without daratumumab. Outcomes: infection-related events, including any-grade infection, grade ≥3 infection, pneumonia, and infection-related death; infections were captured as reported by the trials and, where available, aligned to CTCAE grade definitions (24). Study design: randomized phase II or III controlled trials.

Exclusion criteria comprised observational or single-arm studies, case series, reviews, conference abstracts without full data, duplicate/overlapping reports, and trials not reporting infection outcomes.

### 2.3 Data extraction

Two reviewers independently extracted data using a piloted form, including study characteristics (first author, year, phase, design, country/centers), patient population (NDMM/RRMM), treatment regimens, analysis population (safety vs. intention-to-treat), sample sizes, follow-up duration, and numbers of patients with each infection outcome. Discrepancies were resolved by discussion or by a third reviewer (20). For overlapping publications, the most comprehensive or most recent dataset was used. When necessary, we planned to contact study authors or consult trial registries to clarify unclear data (25).

### 2.4 Risk of bias assessment

Methodological quality was assessed with the Cochrane Risk of Bias tool (RoB 1.0) in Review Manager (RevMan, version 5.4) (26, 27). across seven domains: random sequence generation, allocation concealment, blinding of participants and personnel, blinding of outcome assessment, incomplete outcome data, selective reporting, and other bias. Each domain was judged as low, unclear, or high risk according to the Cochrane Handbook (20). Because most trials were open-label, performance bias was commonly rated as high; outcome-assessment blinding and attrition were judged separately.

### 2.5 Statistical analysis

All quantitative analyses were performed using Review Manager (RevMan, version 5.4; Cochrane Collaboration) (26), and R software (version 4.5.1; metafor package) (28). For dichotomous outcomes, pooled risk ratios (RRs) with 95% confidence intervals (CIs) were estimated using the DerSimonian–Laird random‐effects model; a random‐effects approach was applied regardless of heterogeneity because clinical and methodological diversity was anticipated. Statistical heterogeneity was quantified with I², τ², and Cochran’s Q (29, 30); I² > 50% was interpreted as substantial (31). Predefined subgroup analyses assessed effect modification by disease status (NDMM vs RRMM) and transplant eligibility (TE vs TI), with between‐subgroup differences tested using a test for subgroup differences (interaction p) based on Cochran’s Q (32). Sensitivity analysis consisted of a leave‐one‐out influence analysis, sequentially omitting each trial to evaluate its impact on pooled estimates (33). Zero‐event handling: for trials with zero events in one arm, a continuity correction of 0.5 was applied; trials with zero events in both arms were excluded from that outcome’s meta‐analysis (34). Multi‐arm trials: when multiple daratumumab arms shared a single control, the shared control was evenly split (sample size and events) across comparisons to avoid double counting. Publication bias was assessed by visual inspection of funnel plots in RevMan and, when ≥10 studies were available for an outcome, by Egger’s regression in R (metafor) (20, 35). All tests were two‐sided with p< 0.05 considered statistically significant. For rare-event outcomes, when fewer than three trials reported an outcome and/or when definitions were materially heterogeneous for example, inclusion of COVID-19–related deaths, we prespecified that no meta-analysis would be conducted to avoid unstable or biased estimates; a descriptive synthesis was provided.

## 3 Results

### 3.1 Study selection

**Figure 1:**
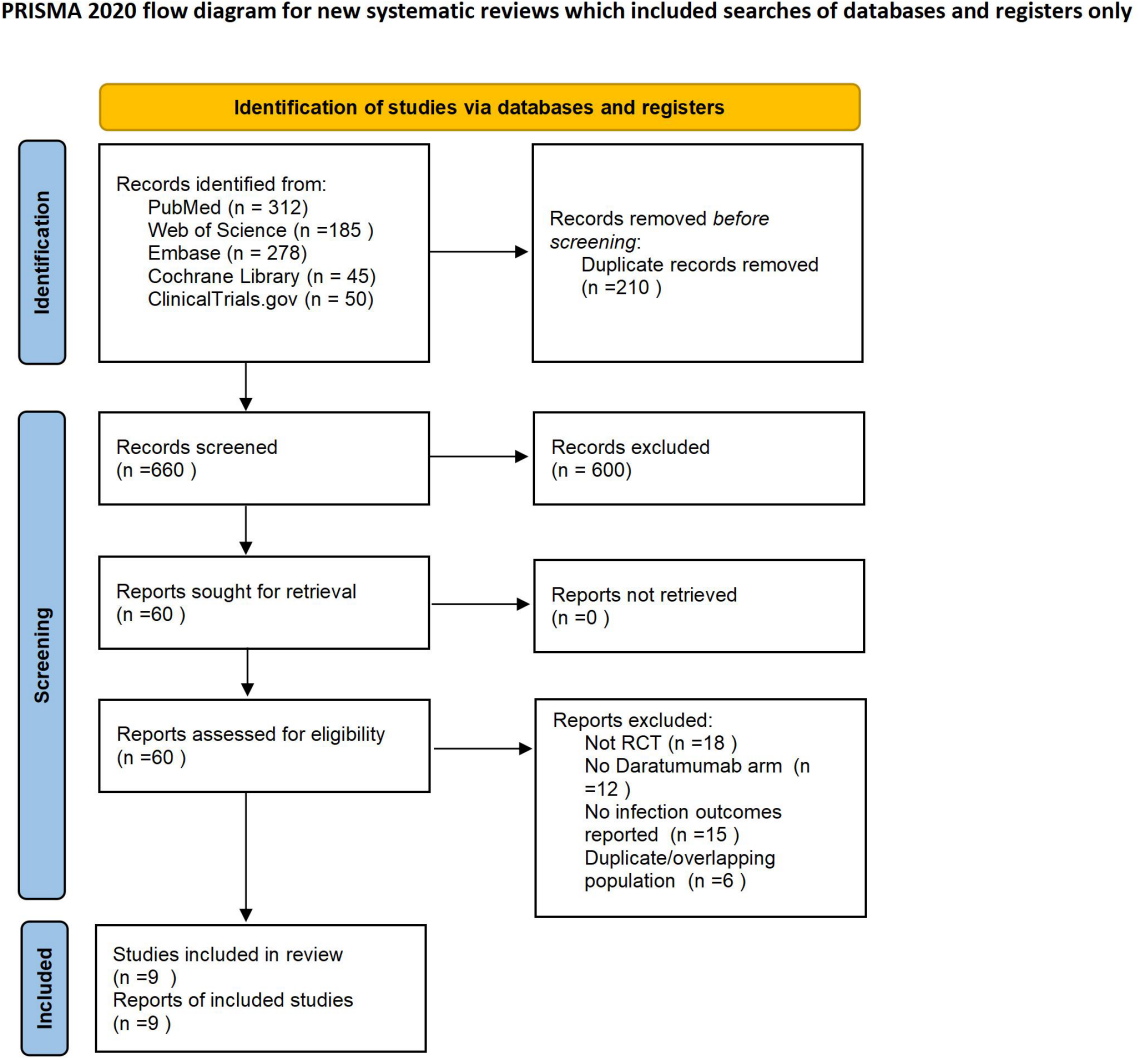
PRISMA 2020 flow diagram of study selection. Flowchart showing the identification, screening, eligibility assessment, and inclusion of randomized controlled trials comparing daratumumab-containing versus standard regimens in multiple myeloma.

A total of 870 records were identified from databases and a trial registry, including 312 from PubMed, 278 from Embase, 185 from Web of Science, 45 from the Cochrane Library, and 50 from ClinicalTrials.gov (20, 21). After removing 210 duplicates, 660 unique records were screened by title and abstract; 600 were excluded for not meeting inclusion criteria. Full-text assessment was performed for 60 articles, of which 51 were excluded for the following reasons: non-randomized design (n = 18), absence of a daratumumab-containing arm (n = 12), no infection outcomes reported (n = 15), or duplicate/overlapping populations (n = 6). Ultimately, nine RCTs met all eligibility criteria and were included in both qualitative and quantitative syntheses. The screening and selection process is shown in **Figure 1** (PRISMA 2020 flow diagram).

The included trials comprised POLLUX (36), CASTOR (15), ALCYONE (37), MAIA (16), CASSIOPEIA (38), GRIFFIN (39), APOLLO (40), CANDOR (41), and PERSEUS (42), covering both newly diagnosed and relapsed/refractory MM populations. Treatment backbones included immunomodulatory drugs (IMiDs), proteasome inhibitors (PIs), and pomalidomide–dexamethasone (Pd).

### 3.2 Study characteristics

Nine RCTs comprising 5,281 patients were included (2,720 receiving daratumumab-containing regimens and 2,561 receiving control therapy). Because reporting of infection endpoints was not uniform across trials, denominators differed by outcome: any infection was available in six RCTs, grade ≥3 infections in all nine RCTs, and pneumonia in nine RCTs (the contributing trials for each endpoint are indicated in the corresponding figures/tables). Key characteristics are summarized in **Table 1**. Expanded baseline demographics and prior lines of therapy are provided in **Supplementary Table S2**. Five trials enrolled NDMM (ALCYONE, MAIA, CASSIOPEIA, GRIFFIN, PERSEUS) (16, 37–39, 42), and four enrolled RRMM (POLLUX, CASTOR, APOLLO, CANDOR) (15, 36, 40, 41). Most studies were phase III; GRIFFIN was a randomized phase II trial (39). Daratumumab was combined with standard backbones including Rd, Vd, VMP, VTd, VRd, or Pd; accordingly, infection outcomes were assessed across IMiD-based, PI-based, and IMiD+PI regimens. Median follow-up ranged from 16 months (APOLLO) to 56 months (MAIA) (16, 40).

**Table 1:** Included randomized trials of daratumumab with baseline characteristics and infection outcomes by treatment arm.

Trial (Author, Year)	Population	Safety N (D/C)	Treatment arms	Age, median (y)	ISS III, %	High-risk cytogenetics, %	Any infection: e/n (%)	Grade ≥3 infection: e/n (%)	Pneumonia: e/n (%)	Infection-related death: e/n (%)
POLLUX (Dimopoulos 2016)	RRMM	286 vs 283	D-Rd vs Rd	65 vs 65	28.6 vs 54.4	15.4 vs 16.6	NR	28.3 vs 22.8	14.1 vs 13.2	NR
CASTOR (Palumbo 2016)	RRMM	251 vs 247	D-Vd vs Vd	64 vs 64	23.5 vs 20.6	22.7 vs 21.3	NR	21.4 vs 19.0	11.9 vs 11.8	NR
ALCYONE (Mateos 2018)	NDMM, TI	350 vs 356	D-VMP vs VMP	71 vs 71	40.6 vs 36.2	16.9 vs 14.9	66.8 vs 48.0	23.1 vs 14.7	15.3 vs 4.8	NR
MAIA (Facon 2019)	NDMM, TI	368 vs 369	D-Rd vs Rd	73 vs 74	38.3 vs 43.6	15.0 vs 13.6	86.3 vs 73.4	32.1 vs 23.3	22.5 vs 12.6	NR
CASSIOPEIA (Moreau 2019)	NDMM, TE	543 vs 542	D-VTd vs VTd	59 vs 58	15.0 vs 15.0	15.0 vs 16.0	65.5 vs 56.9	22.0 vs 19.5	4.0 vs 2.0	NR
GRIFFIN (Voorhees 2020)	NDMM, TE (phase II)	104 vs 103	D-VRd vs VRd	59 vs 61	13.5 vs 13.6	16.3 vs 14.4	90.9 vs 61.8	23.2 vs 21.6	13.1 vs 14.7	NR
APOLLO (Dimopoulos 2021)	RRMM	151 vs 153	D-Pd vs Pd	67 vs 68	22.0 vs 22.0	38.0 vs 32.0	65.1 vs 52.0	24.8 vs 20.0	17.4 vs 11.3	NR
CANDOR (Usmani 2023)	RRMM	312 vs 154	KdD vs Kd	64 vs 64.5	20.0 vs 18.0	15.0 vs 17.0	NR	46.2 vs 32.0	25.6 vs 15.7	1.6 vs 0
PERSEUS (Sonneveld 2024)	NDMM, TE	355 vs 354	D-VRd vs VRd	61 vs 59	15.5 vs 14.2	21.4 vs 22.0	86.9 vs 76.7	35.3 vs 27.4	18.2 vs 11.0	1.1 vs 0.9

Infection outcomes are calculated using the safety population (Safety N). NR, not reported.

### 3.3 Risk of bias assessment

Risk of bias was evaluated using the Cochrane RoB 1.0 tool in RevMan (version 5.4) (26, 27), Overall, the trials demonstrated low risk of bias across most domains (random sequence generation, allocation concealment, incomplete outcome data, and selective reporting). Because most trials were open-label, blinding of participants and personnel was judged at high risk for performance bias; however, blinding of outcome assessment was considered low risk, as infection outcomes were objectively defined and systematically collected per protocol (33). Several studies had minor concerns under “other bias,” mainly related to smaller sample sizes or exploratory design for example, the phase II GRIFFIN trial (39). No major methodological concerns were identified that would materially affect pooled estimates. The overall profile is summarized in **Supplementary Figure S1** (summary plot) and **Supplementary Figure S2** (domain-level distribution).

### 3.4 Any infection

**Figure 2:**
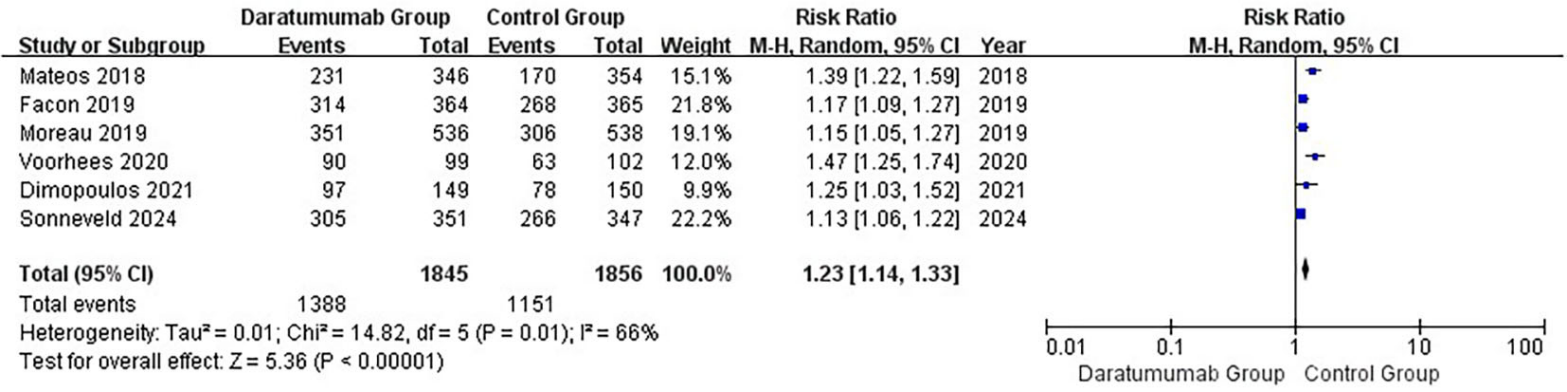
Forest plot of any-grade infection. Pooled analysis of six randomized controlled trials evaluating the risk of any-grade infection with daratumumab-containing versus standard regimens (random-effects model). Results are expressed as risk ratios (RR) with 95% confidence intervals (CIs). Moderate heterogeneity was observed (I² = 66%).

Six RCTs reported any-grade infections (16, 37–40, 42). In the random-effects meta-analysis (**Figure 2**), daratumumab-containing regimens were associated with increased risk of infection (pooled RR, 1.23; 95% CI, 1.14–1.33). Between-study heterogeneity was moderate (I²=66%; τ²=0.01; Q = 14.82, df = 5, p=0.01). The 95% prediction interval was 1.00–1.52, indicating that the true effect in a comparable future setting is expected to fall within this range. Despite heterogeneity, the direction and magnitude of the effect were consistent, and leave-one-out analyses did not materially alter the pooled estimate (33).

### 3.5 Grade ≥ 3 infection

**Figure 3:**
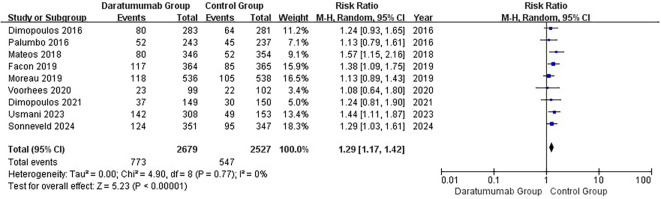
Forest plot of grade ≥3 infection. Nine trials were included. Daratumumab-containing regimens were associated with a higher incidence of severe infection (RR = 1.29, 95% CI 1.17–1.42; p < 0.0001; I² = 0%). The analysis used a random-effects model.

**Figure 4:**
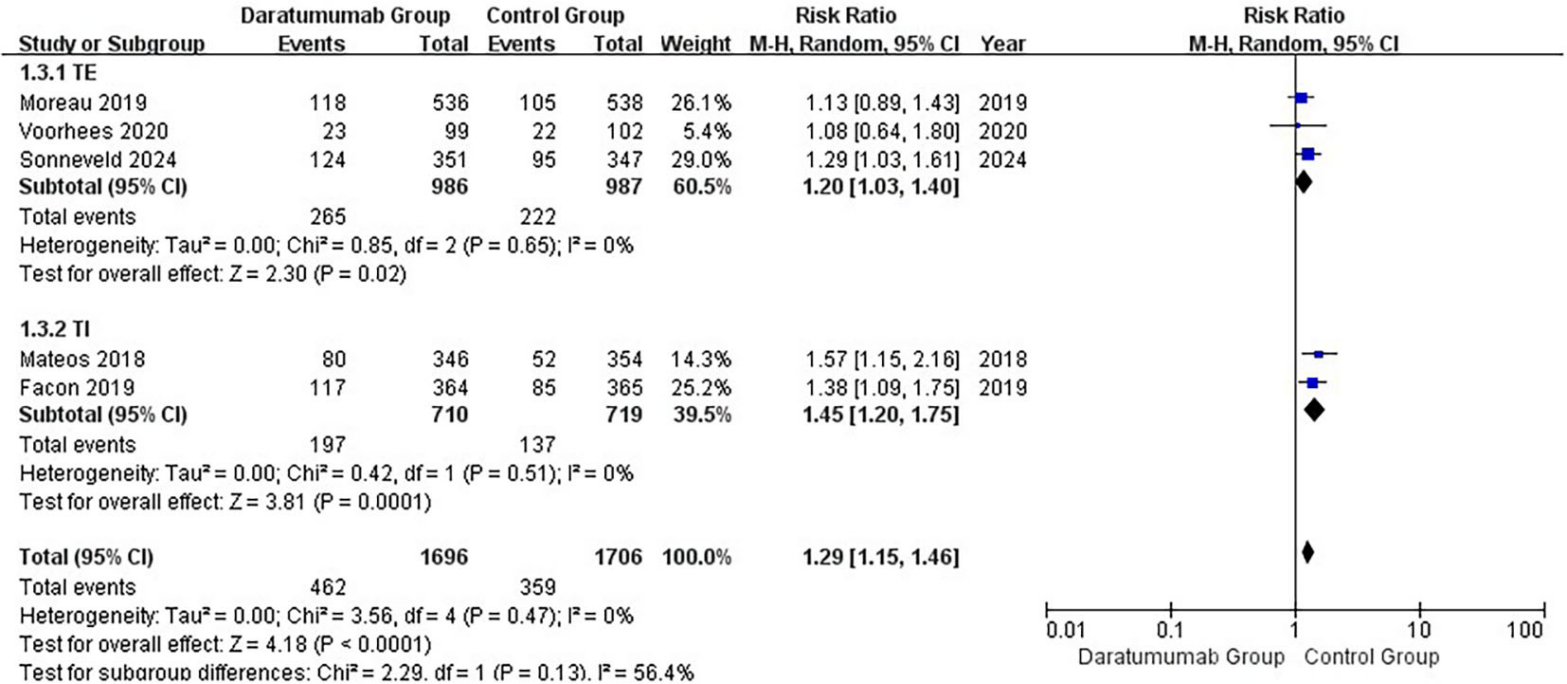
Subgroup analysis of grade ≥3 infection by transplant eligibility (TE vs TI). Random-effects model comparing severe infection risk according to transplant status. No significant interaction was detected between subgroups (p_interaction = 0.13).

All nine RCTs reported grade ≥3 infections (**Figure 3**) (15, 16, 36–42). Daratumumab-containing regimens were associated with a higher incidence of severe infections (pooled RR, 1.29; 95% CI, 1.17–1.42). Between-study heterogeneity was negligible (I²=0%; τ²=0.00; Q = 4.90, df=8, p=0.77). The 95% prediction interval was 1.17–1.42. In subgroup analyses, increased risk was observed in both NDMM (RR, 1.36; 95% CI, 1.18–1.56) and RRMM (RR, 1.23; 95% CI, 1.08–1.40) populations (**Supplementary Figure S3**), with no significant interaction (interaction p=0.30). Similarly, elevated risk was seen in both transplant-eligible and transplant-ineligible groups (TE: RR, 1.20; 95% CI, 1.03–1.40; TI: RR, 1.45; 95% CI, 1.20–1.75; **Figure 4**), again with no significant interaction (interaction p=0.13).

### 3.6 Pneumonia

**Figure 5:**
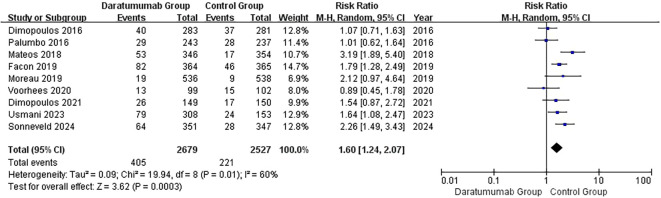
Forest plot of pneumonia. Pooled analysis of eight trials reporting pneumonia events. Daratumumab significantly increased pneumonia risk (RR = 1.60, 95% CI 1.24–2.07; p = 0.0003; I² = 60%).

Nine RCTs reported pneumonia, the most common site-specific infection associated with daratumumab (**Figure 5**) (15, 16, 36–42). Daratumumab-containing regimens were associated with a higher risk of pneumonia (pooled RR, 1.60; 95% CI, 1.24–2.07). Between-study heterogeneity was moderate (I²=60%; τ²=0.09; Q = 19.94, df=8, p=0.01); the 95% prediction interval was 0.84–3.04. In subgroup analyses by treatment backbone, age, and COVID-era timing, point estimates were consistently >1 with no significant between-subgroup interactions (between-subgroup tests: backbone χ²=0.39 [df=2], p=0.82; age χ²=2.25 [df=1], p=0.13; COVID-era χ²=0.00 [df=1], p=0.96; **Supplementary Table S3**). Leave-one-out analysis showed no single trial materially altered the pooled estimate (**Supplementary Table S4**).

### 3.7 Infection-related death

Two trials (CANDOR and PERSEUS) reported infection-related fatalities (40, 41). Given event sparsity, the presence of double-zero trials, and materially heterogeneous definitions (including COVID-19–related deaths), and per our prespecified analysis plan, we did not pool this outcome. Absolute rates were ≤2% in both arms, and no consistent excess mortality with daratumumab was observed across trials.

### 3.8 Sensitivity analyses

A leave-one-out analysis for grade ≥3 infection (**Supplementary Figure S4**) yielded pooled RRs ranging from 1.27 to 1.32, with all 95% CIs > 1.0. The direction and significance of the overall effect were unchanged, and heterogeneity remained low across iterations, supporting the stability of the primary findings (28).

### 3.9 Publication bias

Formal assessment of publication bias was not undertaken because <10 studies contributed to each quantitative outcome (43), limiting the reliability of funnel-plot asymmetry tests (35). Visual inspection of the available effect sizes did not suggest substantial small-study effects or selective reporting.

## 4 Discussion

This systematic review and meta-analysis provides, to our knowledge, the most comprehensive synthesis to date of infection outcomes associated with daratumumab-containing regimens in multiple myeloma. Across nine randomized controlled trials comprising more than 5,000 patients (15, 16, 36–42), daratumumab use was consistently linked to an elevated risk of infection, particularly severe (grade ≥3) infections and pneumonia. The increased risk was evident in both NDMM and RRMM populations, consistent with a potential class effect of anti-CD38 therapy rather than a consequence of treatment stage or backbone regimen.

Our findings extend safety signals reported in individual phase III trials and align with observational analyses showing higher infection rates with daratumumab-based regimens. Mechanistically, the heightened risk likely reflects the broad immunomodulatory effects of CD38 inhibition, most notably depletion of natural killer (NK) cells, reductions in serum immunoglobulin levels, and impairment of humoral immunity, thereby predisposing patients to bacterial and viral respiratory infections (11, 44). This is concordant with the observed increase in pneumonia. Importantly, although infections were frequent, infection-related mortality appeared uncommon: in some large RCTs such as CANDOR, the incidence of fatal adverse events was modest and not clearly attributable to daratumumab, and in the PERSEUS trial safety data described infections including pneumonia and COVID-19 but did not report an excess in mortality (41, 42). These observations suggest that, with vigilant monitoring and supportive care, infection risk remains clinically manageable.

The robustness of the results was supported by leave-one-out sensitivity analysis, which did not materially alter the pooled estimates for grade ≥3 infection (28). Subgroup findings were internally consistent across disease stage and transplant eligibility. For pneumonia, heterogeneity was moderate (I²=60%), likely reflecting differences in patient populations, background regimens, and infection definitions; nevertheless, the direction of effect was consistent across trials. To contextualize relative effects, we quantified absolute risks, risk differences (RD), and numbers needed to harm (NNH) using pooled control risks and random-effects estimates. For any infection, absolute risks were 75.2% with daratumumab vs 62.0% with control (RD + 13.2%, NNH ≈ 8). For grade ≥3 infections, absolute risks were 28.9% vs 21.6% (RD + 7.2%, NNH ≈ 14). For pneumonia, absolute risks were 15.1% vs 8.7% (RD + 6.4%, NNH ≈ 16). These absolute differences complement the relative estimates and support vaccination, antiviral prophylaxis, and IVIG in selected high-risk patients. Collectively, these results underscore the need for systematic infection surveillance and prophylaxis in patients receiving daratumumab. Practical measures include routine monitoring of immunoglobulin levels, timely vaccination (pneumococcal, influenza, and COVID-19), antiviral prophylaxis where appropriate for example, acyclovir for herpes zoster (19), prompt evaluation of febrile or respiratory symptoms, and consideration of intravenous immunoglobulin (IVIG) replacement in patients with recurrent or severe infections and hypogammaglobulinemia (19, 45).

### 4.1 Comparison with previous studies

Previous quantitative syntheses have largely focused on overall safety endpoints or hematologic toxicity rather than infection-specific outcomes (46, 47). By restricting inclusion to phase II/III randomized trials with standardized safety reporting, the present study offers higher-level evidence that more directly quantifies infection risk attributable to daratumumab. Separate analyses of any infection, grade ≥3 infection, and pneumonia further delineate the spectrum and clinical severity of infectious complications.

### 4.2 Limitations

This review has several analytic and reporting limitations. First, our primary random-effects model used the DerSimonian–Laird estimator; alternative approaches such as REML with the Hartung–Knapp–Sidik–Jonkman adjustment (REML-HKSJ) generally yield wider confidence intervals and more conservative inference. In exploratory checks, effect directions were unchanged but precision varied. Second, for rare-event outcomes (notably infection-related deaths), summary estimates may differ by method, for example Peto odds ratio, arcsine risk difference, or continuity-correction models. Given event sparsity, the presence of double-zero trials, and heterogeneous definitions (including COVID-19–related deaths), we prespecified not to pool mortality and instead provided a descriptive synthesis. Third, small-sample issues—specifically zero-event handling (0.5 continuity correction for single-zero trials and exclusion of double-zero trials in some models) and splitting shared controls in multi-arm trials—may introduce modest bias; more complex models (beta-binomial or GLMM) were not applied. Fourth, infection definitions and reporting formats (including different CTCAE versions) varied across trials; we harmonized outcomes at the grade ≥3 threshold and used random-effects models throughout, yet residual heterogeneity likely remains. Fifth, trial-level information on vaccination, routine antimicrobial prophylaxis, and IVIG policies was variably reported and not comparable across studies; these measures were therefore summarized qualitatively and not meta-analyzed. Sixth, study-level aggregation precluded adjustment for individual-level risk factors and prophylactic practices. Seventh, fewer than ten studies contributed to each endpoint, limiting the power and interpretability of funnel plots and Egger’s tests for small-study bias. Eighth, we restricted eligibility to English-language full-text publications and mainly relied on ClinicalTrials.gov for trial registries; consequently, regional or non-English evidence (and non-ClinicalTrials.gov registrations) may have been missed. Finally, most trials predated widespread COVID-19 vaccination and contemporary supportive-care practices, which may affect current absolute risks and the generalizability of pooled estimates.

## 5 Conclusions

In summary, across nine randomized controlled trials, daratumumab-containing regimens were associated with increased risks of infection, particularly grade ≥3 infections and pneumonia, in both NDMM and RRMM settings. In absolute terms, the excess risks were approximately 13% for any infection, 7% for grade ≥3 infections, and 6% for pneumonia, underscoring the need for vaccination, antiviral prophylaxis, and intravenous immunoglobulin (IVIG) where indicated. Infection-related mortality appeared uncommon, and with structured prevention and close monitoring, the overall risk remains clinically manageable as daratumumab use expands to earlier lines and broader combinations.

## Data Availability

The original contributions presented in the study are included in the article/**Supplementary Material**. Further inquiries can be directed to the corresponding author/s.

## Supplementary material

Supplementary Figure 1 Risk-of-bias summary plot (traffic-light format). Visual summary of bias judgments across seven domains for each trial using the Cochrane RoB 1.0 tool.

Supplementary Figure 2 Risk-of-bias domain distribution. Bar chart showing the percentage of trials rated low, unclear, or high risk in each bias domain.

Supplementary Figure 3 Subgroup analysis of grade ≥3 infection by disease status (NDMM vs RRMM). Random-effects model comparing severe infection risk between newly diagnosed and relapsed/refractory populations (p_interaction = 0.30).

Supplementary Figure 4 Sensitivity analysis for grade ≥3 infection (leave-one-out approach). Sequential exclusion of individual trials showing that pooled estimates remained stable (RR range 1.27–1.32; I² low and consistent).

Supplementary Table 1 Detailed database search strategies. Complete search strings for PubMed, Embase, Web of Science, and Cochrane Library, including MeSH terms and keywords.

Supplementary Table 2 Extended baseline and trial characteristics. Trial-level details not shown in **Table 1** (for example, ECOG 0–1%, number of previous lines of therapy by arm, backbone regimen, trial phase, and which infection outcomes were reported).

Supplementary Table 3 Pneumonia subgroup meta-analyses (random-effects). Stratified pooled RRs with 95% CIs for pneumonia by treatment backbone (IMiD vs PI vs IMiD+PI), age (<70 vs ≥70 years), and COVID-era timing (pre-COVID vs during/overlap), with between-subgroup χ² tests.

Supplementary Table 4 Pneumonia leave-one-out sensitivity analysis. Pooled RR (95% CI) after omitting each trial in turn, with corresponding heterogeneity statistics.
