# Supplementary figures and images for: Infection risks associated with daratumumab-containing regimens in multiple myeloma: a systematic review and meta-analysis
Source: Front Oncol. 2026 Jan 6;15:1729177. doi: 10.3389/fonc.2025.1729177 (PMC12815855; doi:10.3389/fonc.2025.1729177)

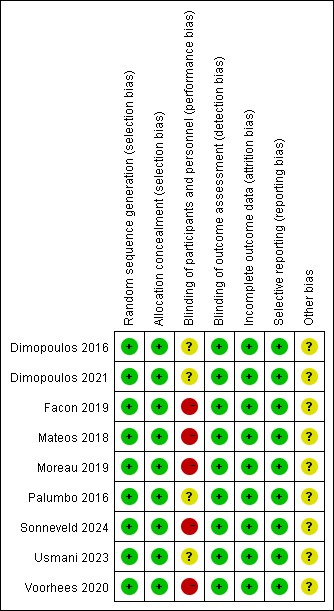

Supplement: Supplementary Figure 1 — Risk-of-bias summary plot (traffic-light format). Visual summary of bias judgments across seven domains for each trial using the Cochrane RoB 1.0 tool. [file Image1.jpeg]

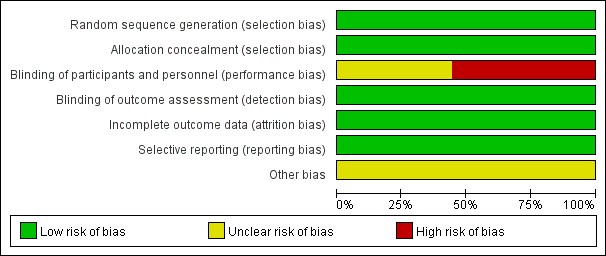

Supplement: Supplementary Figure 2 — Risk-of-bias domain distribution. Bar chart showing the percentage of trials rated low, unclear, or high risk in each bias domain. [file Image2.jpeg]

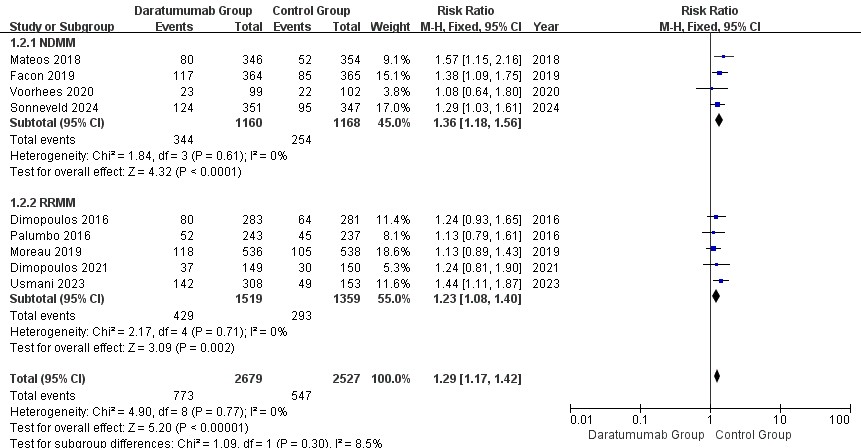

Supplement: Supplementary Figure 3 — Subgroup analysis of grade ≥3 infection by disease status (NDMM vs RRMM). Random-effects model comparing severe infection risk between newly diagnosed and relapsed/refractory populations (p_interaction = 0.30). [file Image3.jpeg]

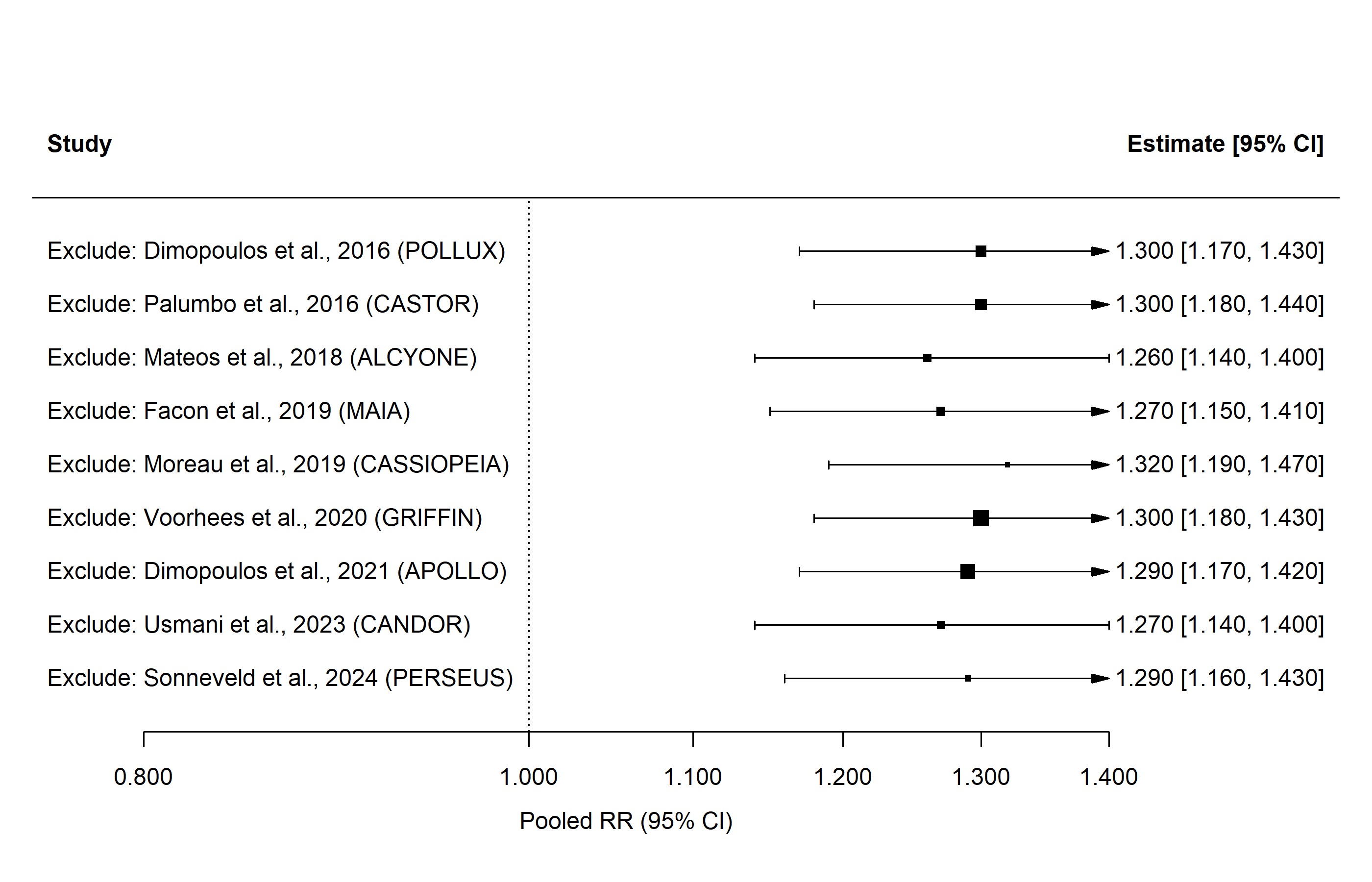

Supplement: Supplementary Figure 4 — Sensitivity analysis for grade ≥3 infection (leave-one-out approach). Sequential exclusion of individual trials showing that pooled estimates remained stable (RR range 1.27–1.32; I² low and consistent). [file Image4.jpeg]
