# Supplementary material for: Infection risks associated with daratumumab-containing regimens in multiple myeloma: a systematic review and meta-analysis
Source: Front Oncol. 2026 Jan 6;15:1729177. doi: 10.3389/fonc.2025.1729177 (PMC12815855; doi:10.3389/fonc.2025.1729177)
Supplement: Supplementary Table 1 — Detailed database search strategies. Complete search strings for PubMed, Embase, Web of Science, and Cochrane Library, including MeSH terms and keywords. [file Table1.docx]

# Supplementary Table S1. Full search strategies for each database

| Database | Search strategy | Filters / Limits |
| --- | --- | --- |
| PubMed | ((("Multiple Myeloma"[Mesh]) OR ("plasma cell myeloma") OR ("Kahler disease") OR ("multiple myeloma"[tiab]))) AND (("Daratumumab"[Mesh]) OR daratumumab[tiab] OR "anti-CD38"[tiab] OR "CD38 monoclonal antibody"[tiab])) AND ((infection[Mesh]) OR infections[tiab] OR pneumonia[tiab] OR "upper respiratory tract infection"[tiab] OR "viral infection"[tiab] OR "bacterial infection"[tiab])) | Publication date: inception to Oct 2025; Language: English OR Chinese; Species: Humans |
| Embase | ('multiple myeloma'/exp OR 'plasma cell myeloma':ab,ti OR 'kahler disease':ab,ti OR 'multiple myeloma':ab,ti) AND ('daratumumab'/exp OR daratumumab:ab,ti OR 'anti cd38 antibody':ab,ti OR 'cd38 monoclonal antibody':ab,ti) AND ('infection'/exp OR infections:ab,ti OR pneumonia:ab,ti OR 'upper respiratory tract infection':ab,ti OR 'bacterial infection':ab,ti OR 'viral infection':ab,ti) | Time: inception–Oct 2025; Language: English or Chinese; Human studies |
| Web of Science Core Collection | TS = (("multiple myeloma" OR "plasma cell myeloma" OR "kahler disease") AND (daratumumab OR "anti-CD38" OR "CD38 monoclonal antibody") AND (infection OR pneumonia OR "upper respiratory tract infection" OR "bacterial infection" OR "viral infection")) | Publication years: All years – Oct 2025; Languages: English or Chinese; Document types: Article, Clinical Trial |
| Cochrane Library (CENTRAL) | ("multiple myeloma" OR "plasma cell myeloma") AND (daratumumab OR "anti-CD38" OR "CD38 monoclonal antibody") AND (infection OR pneumonia OR "respiratory infection" OR "bacterial infection" OR "viral infection") | Trials; no date restriction; English or Chinese |
