# Supplementary material for: Infection risks associated with daratumumab-containing regimens in multiple myeloma: a systematic review and meta-analysis
Source: Front Oncol. 2026 Jan 6;15:1729177. doi: 10.3389/fonc.2025.1729177 (PMC12815855; doi:10.3389/fonc.2025.1729177)
Supplement: Supplementary Table 2 — Extended baseline and trial characteristics. Trial-level details not shown in Table 1 (for example, ECOG 0–1%, number of previous lines of therapy by arm, backbone regimen, trial phase, and which infection outcomes were reported). [file Table2.docx]

# Supplementary Table S2. Included randomized trials: demographics and infection outcomes (daratumumab vs control)

| Trial (Author, Year) | Population | Safety N (D/C) | Treatment arms (D/C) | Age, median (y) | ECOG 0–1, % (D/C) | Number of previous lines of therapy — no. (%) (D/C) | | | Infection outcomes reported | Trial phase | Backbone regimen |
| --- | --- | --- | --- | --- | --- | --- | --- | --- | --- | --- | --- |
|  |  |  |  |  |  | 1 line | 2–3 lines | ≥4 lines |  |  |  |
| POLLUX (Dimopoulos 2016) | RRMM | 286 vs 283 | D-Rd vs Rd | 65 vs 65 | NR | 149(52.1) vs 146 (51.6) | 123(43.0) vs 118 (41.7) | 14(4.9) vs 19(6.7) | ≥3 infection, pneumonia, infection-related death | Phase III | IMiD-based (Rd) |
| CASTOR (Palumbo 2016) | RRMM | 251 vs 247 | D-Vd vs Vd | 64 vs 64 | NR | 122 (48.6) vs 113 (45.7) | 107 (42.6) vs 108 (43) | 22 (8.8) vs 28 (11.3) | ≥3 infection, pneumonia | Phase III | PI-based (Vd) |
| ALCYONE (Mateos 2018) | NDMM, TI | 350 vs 356 | D-VMP vs VMP | 71 vs 71 | 74.3 vs 71.4 | NA | NA | NA | Any infection, ≥3 infection, pneumonia, infection-related death | Phase III | PI-based (VMP) |
| MAIA (Facon 2019) | NDMM, TI | 368 vs 369 | D-Rd vs Rd | 73 vs 74 | 82.9 vs 84.0 | NA | NA | NA | Any infection, ≥3 infection, pneumonia, infection-related death | Phase III | IMiD-based (Rd) |
| CASSIOPEIA (Moreau 2019) | NDMM, TE | 543 vs 542 | D-VTd vs VTd | 59 vs 58 | 90.0 vs 89.0 | NA | NA | NA | Any infection, ≥3 infection, pneumonia, infection-related death | Phase III | IMiD+PI (VTd) |
| GRIFFIN (Voorhees 2020) | NDMM, TE (phase II) | 104 vs 103 | D-VRd vs VRd | 59 vs 61 | 89.1 vs 90.2 | NA | NA | NA | Any infection, ≥3 infection, pneumonia, infection-related death | Phase II | IMiD+PI (VRd) |
| APOLLO (Dimopoulos 2021) | RRMM | 151 vs 153 | D-Pd vs Pd | 67 vs 68 | 96.0 vs 87.0 | (11%) vs 18 (12%) | (75%) 113 vs (74%) | (14%) 22 vs (14%) | Any infection, ≥3 infection, pneumonia, infection-related death | Phase III | IMiD-based (Pd) |
| CANDOR (Usmani 2023) | RRMM | 312 vs 154 | KdD vs Kd | 64 vs 64.5 | 95.0 vs 95.0 | 144 (46) vs 70 (45) | 168 (54) vs 84 (55) | NR | Any infection (respiratory), ≥3 infection, pneumonia, infection-related death | Phase III | PI-based (Kd) |
| PERSEUS (Sonneveld 2024) | NDMM, TE | 355 vs 354 | D-VRd vs VRd | 61 vs 59 | 94.4 vs 95.5 | NA | NA | NA | Any infection, ≥3 infection, pneumonia, COVID-19 infection & death | Phase III | IMiD+PI (VRd) |

*Footnote: Values use the* ***safety population*** *(Safety N). Prior lines are shown as* ***No. (%)*** *with strata* ***1****,* ***2–3****, and* ***≥4*** *(NDMM =* ***NA****).* ***NR****, not reported;* ***NA****, not applicable;* ***D/C****, daratumumab-containing/control arm.*
