# Supplementary material for: Infection risks associated with daratumumab-containing regimens in multiple myeloma: a systematic review and meta-analysis
Source: Front Oncol. 2026 Jan 6;15:1729177. doi: 10.3389/fonc.2025.1729177 (PMC12815855; doi:10.3389/fonc.2025.1729177)
Supplement: Supplementary Table 3 — Pneumonia subgroup meta-analyses (random-effects). Stratified pooled RRs with 95% CIs for pneumonia by treatment backbone (IMiD vs PI vs IMiD+PI), age (<70 vs ≥70 years), and COVID-era timing (pre-COVID vs during/overlap), with between-subgroup χ² tests. [file Table3.docx]

**Supplementary Table S3. Pneumonia subgroup meta-analyses (random-effects)**

| Stratification | Subgroup | k (trials) | Events D/C | N (Safety) D/C | Pooled RR (95% CI) | Heterogeneity (I², τ², Q p) |
| --- | --- | --- | --- | --- | --- | --- |
| Backbone | IMiD-based | 3 | 148 / 100 | 796 / 796 | **1.45 (1.05–2.01)** | I²=44%, τ²=0.04, Q(2)=3.57, p=0.17 |
|  | PI-based | 3 | 161 / 84 | 897 / 744 | 1.73 (0.94–3.18) | I²=80%, τ²=0.23, Q(2)=9.98, p=0.007 |
|  | IMiD+PI | 3 | 96 / 52 | 986 / 987 | 1.67 (0.93–2.99) | I²=62%, τ²=0.16, Q(2)=5.28, p=0.07 |
| Age (trial-level median) | <70 years | 7 | 270 / 158 | 1969 / 1775 | **1.42 (1.09–1.86)** | I²=48%, τ²=0.06, Q(6)=11.62, p=0.07 |
|  | ≥70 years | 2 | 135 / 63 | 710 / 719 | **2.30 (1.31–4.05)** | I²=70%, τ²=0.12, Q(1)=3.37, p=0.07 |
| COVID-era | Pre-COVID (≤2019) | 5 | 223 / 137 | 1772 / 1775 | **1.63 (1.08–2.45)** | I²=73%, τ²=0.15, Q(4)=14.57, p=0.006 |
|  | During/overlap (2020–2024) | 4 | 182 / 84 | 907 / 752 | **1.61 (1.15–2.25)** | I²=43%, τ²=0.05, Q(3)=5.27, p=0.15 |

**Footnotes.** Random-effects model (DerSimonian–Laird); effect size is the risk ratio for pneumonia. “N (Safety)” denotes the per-arm safety population used for infection endpoints. To maintain concision, 95% prediction intervals (PIs) were reported for the primary pneumonia analysis in the main text; subgroup PIs are not shown due to the small number of trials per subgroup but are available upon request.
**Between-subgroup tests (Cochran’s Q).** backbone χ²=0.39 (df=2), p=0.82; age χ²=2.25 (df=1), p=0.13; COVID-era χ²=0.00 (df=1), p=0.96.
