# Supplementary material for: Infection risks associated with daratumumab-containing regimens in multiple myeloma: a systematic review and meta-analysis
Source: Front Oncol. 2026 Jan 6;15:1729177. doi: 10.3389/fonc.2025.1729177 (PMC12815855; doi:10.3389/fonc.2025.1729177)
Supplement: Supplementary Table 4 — Pneumonia leave-one-out sensitivity analysis. Pooled RR (95% CI) after omitting each trial in turn, with corresponding heterogeneity statistics. [file Table4.docx]

# **Supplementary Table S4. Leave-one-out sensitivity analysis for pneumonia (random-effects model)**

| Trial omitted | Acronym | Backbone | Pooled RR  (95% CI) | I² | τ² | χ²  (df=7), p |
| --- | --- | --- | --- | --- | --- | --- |
| Dimopoulos 2016 | POLLUX | IMiD (Rd) | **1.70 (1.31–2.21)** | 55% | 0.07 | 15.56, 0.03 |
| Palumbo 2016 | CASTOR | PI (Vd) | **1.70 (1.31–2.20)** | 56% | 0.07 | 15.93, 0.03 |
| Mateos 2018 | ALCYONE | PI (VMP) | **1.48 (1.18–1.87)** | 46% | 0.05 | 12.87, 0.08 |
| Facon 2019 | MAIA | IMiD (Rd) | **1.57 (1.16–2.13)** | 64% | 0.12 | 19.53, 0.007 |
| Moreau 2019 | CASSIOPEIA | IMiD+PI (VTd) | **1.57 (1.19–2.06)** | 64% | 0.10 | 19.46, 0.007 |
| Voorhees 2020 | GRIFFIN | IMiD+PI (VRd) | **1.69 (1.30–2.18)** | 59% | 0.08 | 16.92, 0.02 |
| Dimopoulos 2021 | APOLLO | IMiD (Pd) | **1.61 (1.21–2.14)** | 65% | 0.10 | 19.91, 0.006 |
| Usmani 2023 | CANDOR | PI (Kd) | **1.60 (1.19–2.15)** | 65% | 0.11 | 19.95, 0.006 |
| Sonneveld 2024 | PERSEUS | IMiD+PI (VRd) | **1.52 (1.16–2.00)** | 59% | 0.09 | 17.10, 0.02 |

**Overall (all 9 trials):** RR **1.60 (1.24–2.07)**; I² **60%**; τ² **0.09**; χ² **19.94** (df=8), p = 0.01.

**Footnotes:**
RR = risk ratio; CI = confidence interval. Random-effects model (Mantel–Haenszel, DerSimonian–Laird). Outcome: pneumonia. Leave-one-out analysis excludes one study at a time (8 studies remaining per row). Backbones: Rd = lenalidomide–dexamethasone; Vd = bortezomib–dexamethasone; VMP = bortezomib–melphalan–prednisone; VTd = bortezomib–thalidomide–dexamethasone; VRd = bortezomib–lenalidomide–dexamethasone; Pd = pomalidomide–dexamethasone; Kd = carfilzomib–dexamethasone.
